# Development of a serotype colloidal gold strip using monoclonal antibody for rapid detection type Asia1 foot-and-mouth disease

**DOI:** 10.1186/1743-422X-8-418

**Published:** 2011-09-01

**Authors:** Tong Lin, Jun-jun Shao, Jun-zheng Du, Guo-zheng Cong, Shan-dian Gao, Huiyun Chang

**Affiliations:** 1 State Key Laboratory of Veterinary Etiological Biology, National Foot and Mouth Disease Reference Laboratory, Lanzhou Veterinary Research Institute, Chinese Academy of Agricultural Sciences, Lanzhou 730046, China

**Keywords:** Foot-and-Mouth disease virus (FMDV), type Asia1, Monoclonal antibodies, Colloidal gold strip, RIHA assay

## Abstract

**Background:**

In this study, we developed a rapid, one step colloid gold strip (CGS) capable of specifically detecting type Asia1 foot-and-mouth disease virus (FMDV). We have produced two monoclonal antibodies (mAb) to type Asia1 FMD (named 1B8 and 5E2). On the test strip, the purified 1B8 labelled with the colloidal gold was used as the detector, and the purified 5E2 and goat anti-mouse antibodies were wrapped onto nitrocellulose (NC) membranes as the test and the control line, respectively. The rapid colloidal gold stereotype diagnostic strip was housed in a plastic case.

**Results:**

In specificity and sensitivity assay, there was no cross-reaction of the antigen with the other type of FMD and SVDV. The detection sensitivity was found to be as high as 10^-5 ^dilution of Asia1/JSL/05 (1 × 10^7.2^TCID_50_/50 μL). There was excellent agreement between the results obtained by CGS and reverse indirect hemagglutination assay (RIHA), and the agreement can reach to 98.75%.

**Conclusion:**

We developed colloidal gold strips that have good qualities and does not require specialized equipment or technicians. This method provided a feasible, convenient, rapid, and effective for detecting type Asia1 FMDV in the fields.

## Background

All cloven-hoofed species are susceptible to foot-and-mouth disease (FMD), and this disease is characterize by fever, vesicular lesions and erosion in the mouth and on the tongue, muzzle, feet and teats and cause great economic losses in the affected countries and they involve an extensive threat for rapid and wide spread [1]. FMDV as pathogeny of FMD is a member of the family *Picormaviriae *and exists in seven immunological distinct serotypes (Asia1, A, O, C, SAT1, SAT2 and SAT3). Incidence of type O, A and C has been recorded in different parts of the world, however, incidence of types Asia1 and SAT1 to 3 is mainly restricted to Asia1 and Southern Africa, respectively [2].

During the Aisa1 type FMD outbreak in the China in 2005, the requirement for a 24 h slaughter policy did not allow sufficient time for laboratory confirmation of suspect infection following clinical diagnosis. A rapid test or field-based assay would be a valuable tool to initial diagnosis of FMDV in a suspect animal. Many of sensitive methods such as RIHA and RT-PCR have been developed to analyze FMDV in nasal swabs, epithelial suspensions and probangs of clinical samples submitted from the field or animals infected experimentally with cell culture [3,4]. Thus, it is extremely desirable to develop a rapid and convenient detection method for FMDV.

The convenience and speed of the test have been achieved by a novel concept of immunochromatographic (IC) assay, which depends on the transport of tag (usually is colloidal gold)-labeled antibody (or antigen) probe and its binding partner-specific antigen (or antibody) immobilized on the surface of the membrane. The transfer is induced by the capillary action of aqueous medium through membrane pores to separate the unbound reactant from the bound complex at the liquid-solid interface. Among different tags/marks-labeled test systems, colloidal gold appears to be most attractive [5]. The application of this technology in the field of animal medicine starts latter than others, and the reported researches of FMD rapid diagnosis technology mainly refer to the diagnosis of FMD antibody[6]. And qualitative pathogenicity[7,8], however, the report on the GICA applied for the multi-serotype diagnosis for the cause of FMD is infrequent. This research is directly towards to the complicated operation of nowadays FMDV stereotype technology, which is difficult to use to diagnose the FMDV pathogen in the grass-roots and field pathogens, and as the same time according to in Asia or China FMDV epidemic status, FMDV serotype O, A, Asia1was more frequently found. Therefore, we developed a new, one-step confirmatory test based on an immunochromatographic assay for serotype detection of FMDV.

## Materials and methods

### Cell and virus

Reference FMDV O/CHA/99, A/GS/LX/62, Asia1/JSL/05 and swine vesicular disease virus (SVDV) were preserved by the National Foot-and-mouth Disease Reference Laboratory of China. Field specimens (including epithelial suspensions prepared from current and historical field samples and virus isolation in cell and souking mouse culture) were provided by National Foot-and-mouth Disease Reference Laboratory of China. SP2/0 cell were purchased from ATCC (Manasa, VA) and was cultured in RPMI-1640 (Sigma, UK) supplement with 10% fetal calf serum.

### Mice immunization and mAb production

Female BALB/C mice of 5-6 weeks old was immunized with 10-20 mg of inactivated FMDV type Asia1 antigen in an equal volume of complete Freund's adjuvant subcutaneously. Three identical boosters emulsified in incomplete Freund's adjuvant were given at 3 weeks interval. Mice were boosted with the same antigen in PBS by intraperitoneal injection 3-4 days before cell fusion. RPMI -1640 with 10% fetal bovine serum was used for fusion and subclone. Immunized spleen cells were fused with myeloma cells at 5 to 10:1 ratio in the presence of 50% polyethylene glycol 1500 (MERCK). The cells were plated out in semisolid medium (Stem Cell) and incubated at 37°C in humidified 5% CO2 atmosphere (Davis et al., 1982). After 7 to 10 days, single colonies were transferred to 96-well culture plates. Hybridoma supernatants were screened using ELISA. The positive hybridomas were subcloned using limiting dilution technique. The mAb isotyping was performed using a mouse monoclonal antibody isotyping kit (Isostrip, Roche) according to manufacturer's instruction. The specificity, stability, titers and neutralization activity were described as previously [9].

### Purification of monoclonal antibody

The crude extract of prepared monoclonal antibody (mAb) were obtained by ammonium sulfate, and further purified by DEAE-Sepharoseion exchange chromatography column [10]. The quantification of protein was measured by SDS-PAGE [11]. And the double-phase immunodiffusion method measured titer of purified antibody [12].

### Preparation of colloidal gold

5 ml of a 1% (w/v) stock solution of hydrogen tetrachloroaurate trihydrate was added to 500 ml solutions of distilled water and heated to the boiling point, and then 5 ml of freshly made 1% solution of sodium citrate were added to the gold solutions under constant stirring. The mixture was boiled until it turned red. After an additional 5 min of boiling, then the solution was stored at 4°C.

### Monoclonal Antibodies labeling

The optimal concentration of mAb for conjugation with colloidal gold was determined by titrating aliquots of diluted IgG with colloidal gold. The purified IgG was diluted to a concentration of 0.1 mg/ml in phosphate buffered solution (0.002 M, pH 9.0). The pH of colloidal-gold solution and the diluted IgG was adjusted to pH 8.3 with 0.1 M K_2_CO_3_. Ten aliquots of variable concentrations (0.01-0.1 mg/ml) of the diluted IgG were prepared in 0.2 ml phosphate buffer, and added separately to 1 ml of the colloidal-gold solution. After incubating the mixture for 10 min, 0.1 ml of 10% NaCl was added to the tubes and the absorbance was measured at 520 nm. The least amount of protein required to stabilize the colloidal gold was identified from the abscissa in the curve drawn from the concentration and the absorbance. Aliquot (7-10 ml) of purified IgG (0.1 mg/ml) was added drop-wise to l00 ml of colloidal-gold solution (pH 9.0) followed by the addition of 10 ml of filtered 10% BSA, pH 9.0 with gentle stirring for 20-25 min. The solution was incubated for 1 h at 4°C and centrifuged at 15,000 g for 30 min at 4°C. The supernatant was discarded and the loose precipitate of gold conjugate was re-suspended in 5 ml conjugate dilution buffer (0.01 M Tris, 5% BSA, 2% sucrose, 0.87% NaCl and 0.1 M sodiumazide) and stored at 4°C.

### Preparation of colloid gold strip

The pH of the colloidal gold solution was adjusted to pH around 7.4 before the mAb was added. This was used in as much as the laboratory experience supports the optimum pH at 7-8 for labeling mAb with colloidal gold. The least concentration of mAb for stabilizing colloidal gold particles is on a basis of 0.03 mg/ml for the above experimentation. Therefore, 2.5 ml mAb (0.04 mg/ml prepared in pH 7.4, PBS 0.01 mol/L) was added drop-wise to 2.5 ml of colloidal gold solution. The solution was stored for 2 h at 4°C and then was centrifuged to remove unconjugated mAb, followed by the pellet re-dispersed in total 5 ml of pH 7.4 PBS and stored at 4°C for further experiments. The formation of antibody-colloidal gold conjugation was monitored with UV is spectroscopy using a double-beam spectrophotometer (Lambda 25, Perkin Elmer Instruments, USA).

Immunochromatography strip was constructed as a method by Xu et al. [13] and Verheijen et al. [14]. Colloidal gold-labeled antibody conjugate was jetted onto glass fiber and dried at 37°C. Goat anti-mouse antibody (1.0 mg/ml) was dispensed onto a nitrocellulose membrane on the upper line for control with a volume of 1 μl per 1 mm line, and for another epi-position strain mAb (1.0 mg/ml) in PBS was jetted into the lower part for test line; the dispensed volume was also of 1 μl per 1 mm line. The remaining active sites on the membrane were blocked by incubation with 2% BSA in PBS (1 ml/cm membrane) for 30 min at room temperature. The membrane was washed once with PBS and again with distilled water and then, dried at 37°C. Subsequently, the absorption pad, the nitrocellulose membrane, the released antibody-gold conjugation pad, and the sample application pad were assembled into a sheet of plastic backing orderly and cut into individual strips (2.0 mm/strip) with a pair of small medicine scissors.

### Detection principle and procedure for the colloid gold strip

In the detection test, the sample is allowed to react with the colloidal gold-FMDV mAb conjugate. The mixture then moves upward on the nitrocellulose membrane chromatographically by the capillary action. For a positive sample, the conjugate binds to the antigen forming a gold-antigen-FMDV mAb complex, which binds to staphylococcal protein A (SPA) and forms a red color band at the test region. Absence of this band suggests a negative result. To serve as a procedural control, a red band at control region will always appear regardless of the presence of FMDV antigen. The samples were firstly diluted 1:50-1:100 with PBS and placed in a well on a 96-well plate, respectively. The strip was dipped into the solution for 10-20 s, then taken out and placed horizontally for 1-2 min to observe the result. If both the test and control lines turn red, the sample is recorded as positive. When the control line but not the test line was colored, it is considered negative.

### Specificity and sensitivity of the strip

The specificity of the strip test was evaluated with standard negative samples, standard positive samples and the pathogens for FMDV O, A type and SVDV. Approximately 100 μl of each sample was added to the sample chamber and let stand for 15 min. A result was considered positive when red-purple bands appeared at both the test line and the control line. A result was considered negative when a red-purple band only appeared at the control line.

The sensitivity of the strip test was tested with serially diluted type Asia1 FMDV reference positive cell culture virus (Asia1/JSL/05 1 × 10^7.2^TCID_50_/50 μL). The positive antigen with a ten fold serial dilution was diluted with PBS from 10^-1 ^to 10^-9^. The diluted antigen was tested with this strip. The same procedure was repeated three times with different operators.

### Examination of samples collected in the field

FMDV serotype field specimens stored by LVRI national FMD reference laboratory, all samples were tested by RIHA to characterize the specificity of the virus serotype in the original material. Furthermore, the virus isolation test and RT-PCR/nucleotide sequencing were used to validate the serotype of the samples (data not shown). The results of the RIHA were compared with CGS.

## Results

### Prepared mAbs

**Figure 1:**
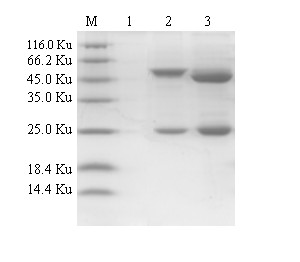
**Analysis of purified mAbs**. Lane M is protein molecular weight Marker; Lane 1 is negative control; Lane 2 is mAb 1B8; Lane 3 is mAb 5E2.

The prepared mAbs 1B8 and 5E2 were target for different epitope of FMDV VP1 protein, and all of them belonged to IgG1 isotype [9]. Purified mAbs was analyzed with SDS-PAGE, showing a light chain (25 KDa) and a heavy chain (50 KDa), which was consistent with the predicted molecular weight(Figure 1). And these mAbs specifically reacted to VP1 antigen of FMDV type Asia 1(data was not shown).

### Test on colloidal gold evenness

Control the concentration by adding certain distilled water to the cooled colloidal gold, and the detection wavelength of colloidal gold solution qualified is in range 1.1~1.2 OD_523 nm_. The size of the particle of the prepared colloidal gold diameter the measured average size is 40.06 ± 0.7 nm (*n *= 250) by using the transmission electron microscope. More than 100 particles were measured and variation coefficient CV of these particles is 9.6% (data was not shown).

### Immunochromatographic test

**Figure 2:**
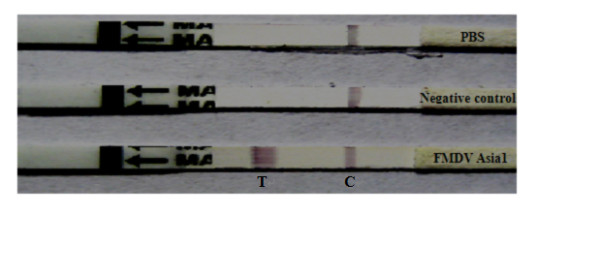
**Test results of CGS validation**. FMDV Asia 1 results were judged by the appearance of two lines in the control and test lines. Negative control and PBS results were judged by the appearance of only a single color line in the control line.

The system for the immunochromatographic assay was optimized by determining the physical and chemical conditions for maximum sensitivity of measurement. Various concentrations of recombinant core protein were assayed by the core test strip. Analysis was complete in less than 15 min. A positive result was indicated by the appearance of two red lines in the control and test lines, and a negative result was indicated by the appearance of only a single line in the control line. An inconclusive result was indicated by a weak red color in the test regions [15]. This CGS kit can serotype detect FMDV Asia 1 type, and no cross reacted to FMDV other type. If the test strip gave a positive result, this indicated that FMDV serotype Asia 1 was present in the sample (Figure 2)

### Specificity and sensitivity experiment

**Figure 3:**
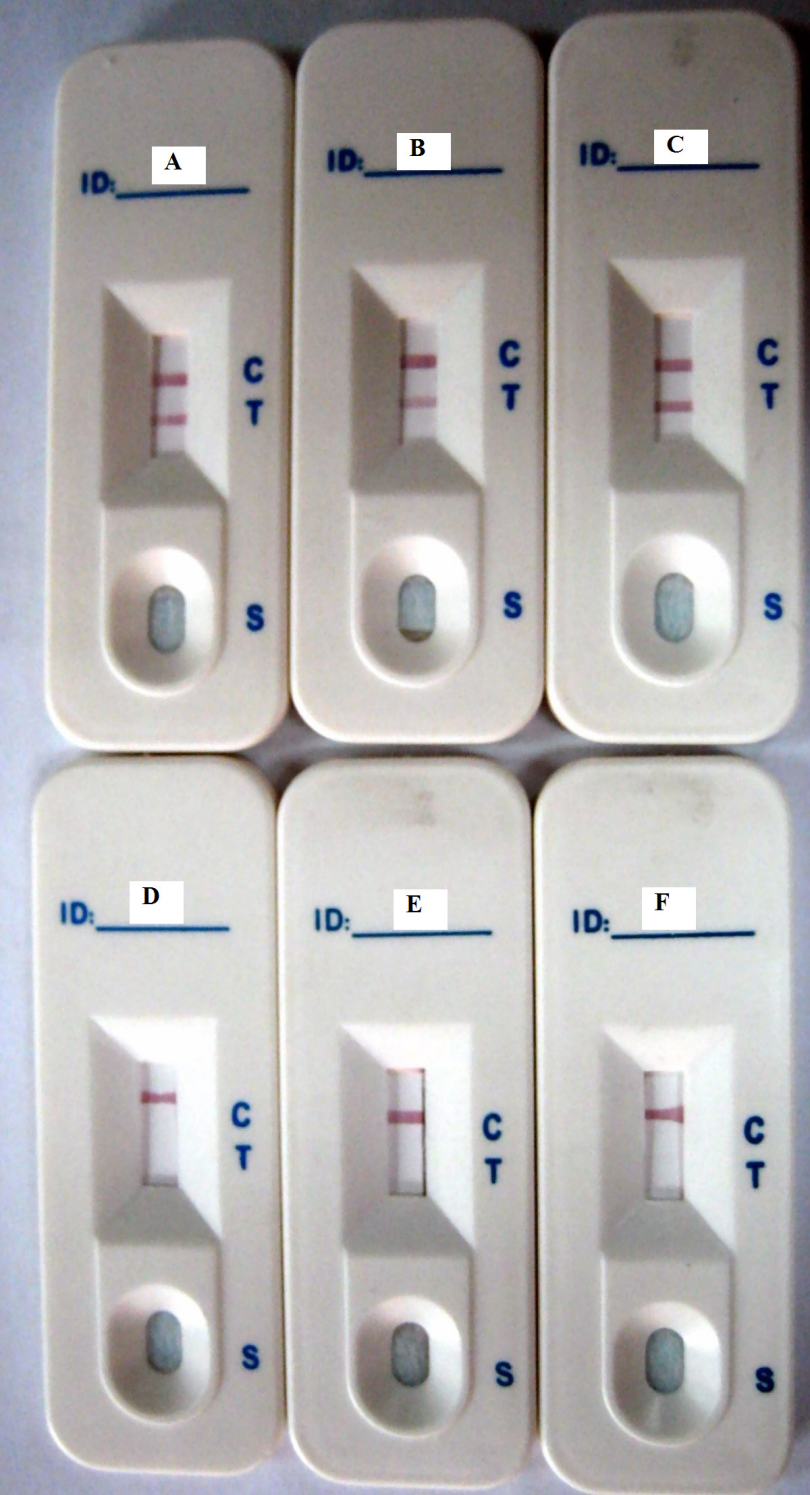
**Test results of specific assay of the strip**. A is CGS detect Asia 1 type FMD cell virus; B is CGS detect Asia 1 type FMD suckling mouse virus; C is CGS detect VP1 protein of Asia 1 type FMDV; D is CGS detect A type FMD cell virus; E is CGS detect O type FMD cell virus; F is CGS detect SVDV.

The specificity of the strip was evaluated in comparison to other virus (including FMDV serotype A, O and SVDV). As shown in Figure 3, two clear red lines were observed in the test and the control lines for detecting type Asia1 FMDV (including cell virus, suckling mouse virus and recombinant VP1 protein), but just only one red control line was observed in detecting other type FMDV (O and A) and SVDV. This indicated that the developed strip had a high specificity to type Asia1 FMDV without cross reaction for other virus.

**Figure 4:**
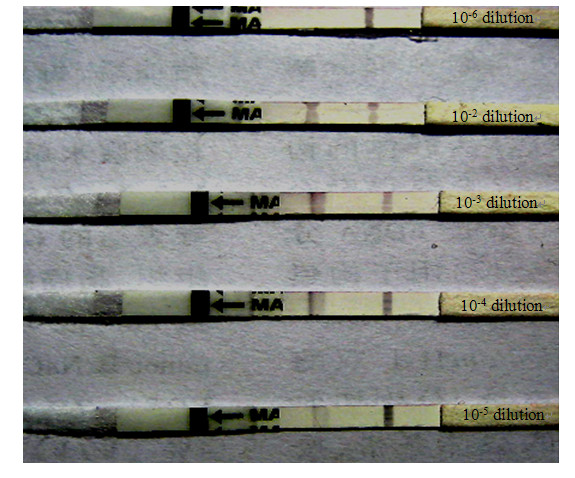
**Test results of sensitive assay of the strip**.

The sensitivity of the strip was tested with type Asia1 FMDV reference positive cell culture virus (Asia1/JSL/05) diluted serially, two red bands developed at the test line with a highest dilution of 10^-5^, but just only control line appeared in dilution reach to 10^-6 ^(Figure 4). This indicated that the strip has a high sensitivity for detecting small amount of FMDV type Asia1 virus. The same results were also repeated by other operator.

### Detection in field specimens

To assess the use of the strips for the detection of FMDV in field, 96 samples were describe as previously, every sample was detected by prepared strip and commercial RIHA at same time. The agreement of the test strip with the RIHA was 98.75%. This indicated that the two methods show good correspondence (Table 1)

**Table 1:** Comparison of the CGS with RIHA

Type of sample	Number	Asia1 RIHA		Asia1 CGS	
		
		Positive	Negative	Positive	Negative
O	20	0	20	0	20
A	2	0	2	0	2
Asia1	18	16	2	17	1
Negative sample	56	0	56	0	56
Total	96	16	80	17	79

## Discussion

With the mutations of foot-and-mouth disease virus, the new variant strains and subtypes come out ceaselessly [16]. It is extremely difficult to prevent and control the FMD. The laboratory diagnoses of the suspected FMD specimens are very crucial to accurately grasp the epidemic situation of FMDV and develop effective precautionary measures. As for the diagnosis of FMD, the specimens should be collected from the fresh blister skin and fluid which is not ulcerated and damaged in the epidemic areas, and then transported to the specialized laboratories for diagnosis. On the one hand, in the developing countries, the uncertain factors in the transportation often lead to the putridity of specimens, false negative, omission of test, etc, thereby to bungle the opportunity to clarify a diagnosis; on the other hand, the existing improper preservation of the specimens shall bring on a risk of virus spreading. However, among the current diagnostic methods of FMD, the methods such as virus isolation, neutralization tests, indirect ELISA, RT-PCR, etc. must be completed in the laboratories on certain conditions. In current status, the grass-roots inspection institutions and vast livestock and poultry farms in China, it is lack of detecting apparatus and specialized technical personnel. So it becomes urgent to develop a rapid and simple new diagnostic method which can be operated on the wild and field without professionals and equipments.

The FMDV consists of seven serotypes and multiple subtypes within each serotypes. The majority of polyclonal and monoclonal antibodies against FMDV produced were serotype specific. In this study, FMDV Asia1 whole virus was used for immunization. The cross-reactions of mAbs are due to two common sites on different serotypes of FMDV. The mAbs, 1B8 and 5E2 are able to detected FMDV in infected tissue and cell because the mAb recognizes the native epitope [9]. The prepared test paper strip can identify FMDV Asia 1 type and differ from other type of FMDV (including type O and A) and SVDV.

This kit mainly adopts pandemic strains of mAbs as the capture antigen of colloidal gold pad and the test antigen of NC membrane. It will increase the dominant epitope recognized by antibody to the combination of capture of test antigen and specificity thought the application of the serotype of strains, extent the screening range of the spectrotype of homotype strains, improve the accuracy and sensitivity of this method, and reduce the omission factor.

Generally, GICA use staining emulsion and colloidal gold as the makers. The staining emulsion particles are larger than colloidal gold particles [17]. In the chromatography test, the NC membrane can accommodate a large amount of particles. The smaller the diameters of the particles are the better degree of mixing can be achieved by the adsorption line along the membrane, so that it can improve the sensitivity of detection. In addition, the tiny particles can be deposited compactly at the adsorption line to get better identification. At the same time, the colloidal gold particles can adsorb protein antibodies initiatively. This combining proves is relatively simple. It means there is no requirement for other reagents, in addition to protein, dilution buffer and gold particles. But the staining emulsion is required to treat specially in order to connect with the covalent bonds of protein. Thus, compared with staining emulsion, the colloidal gold is more suitable to be as the maker of rapid detection. Recently, all of new makers used in rapid detection [18] are the colloidal gold particles.

In the colloidal gold immunochromatography test, the 40 mm particles are preferred [19]. The particles with this size are proper and are easy to be identified. Moreover, it will not affect the combination of the protein and the particles, so the marked materials can achieve the best performance. This experiment use the colloidal gold prepared with the magnetic stirring. Which make the average colloidal gold particle diameter is 40.06 ± 0.7 nm. In addition, the shapes of the particles are uniform, and the coefficient of dimensional variation is small. Therefore, the particles seldom agglutinate can flow on the NC membrane easily.

The clinical symptoms of FMD are very similar with other vesicular disease, especially SVDV. Therefore, it is required to test these pathogens together with FMDV by the specificity experiment. The results of the test kit indicated that the specimens of non-Asia1 type FMDV was negative, the coincidence rates of FMD serotype is 98.75% (Table 1). It is concluded that there is no cross and false positive reactions with other viruses by using this kit. So the specificity of this kit is excellent.

## Conclusions

The present rapid diagnosis kit should enable to detect Asia1 FMDV from epithelial suspension and the virus isolation in cell and souking mouse culture. The kit shows good sensitivity, specificity, reproducibility and stability. It has got good feedback and evaluations in many provinces in China which shows the application and commercial prospect of the kit.

## Competing interests

The authors declare that they have no competing interests.

## Authors' contributions

TL participated in the prepared mAbs, CGS and drafted the manuscript. JD carried out the CGS. GC and SG participated in the design of the study and performed the statistical analysis. XL and HC conceived of the study, and participated in its design and coordination. All authors read and approved the final manuscript.
